# Do self-help groups improve sexual and reproductive health and HIV outcomes among female sex workers in sub-Saharan Africa? A scoping review protocol

**DOI:** 10.12688/wellcomeopenres.23002.1

**Published:** 2024-09-19

**Authors:** Gracious Madimutsa, Fortunate Machingura, Owen Nyamwanza, Frances Mary Cowan, Webster Mavhu

**Affiliations:** 1 Liverpool School of Tropical Medicine, Liverpool, England, UK; 2 Centre for Sexual Health and HIV/AIDS Research Zimbabwe, Harare, Harare Province, Zimbabwe

**Keywords:** Scoping review protocol; Female sex worker; Sub-Saharan Africa; Self-help group; HIV; sexual reproductive health

## Abstract

**Introduction:**

Self-help groups (SHGs) have been effective in improving the health and wellbeing of women yet there is a dearth of evidence on how they can improve female sex workers’ (FSWs) HIV and sexual and reproductive health (SRH) outcomes, particularly in sub-Saharan Africa (SSA). The proposed scoping review seeks to address this gap by identifying and analysing literature on SHGs for FSWs in SSA.

**Materials and methods:**

This scoping review will employ the methodology developed by Arksey and O’Malley (2005), expanded on by Levac and colleagues (2010) and Colquhoun and colleagues (2014), and further outlined by Peters and colleagues (2020): (1) identifying the research question(s); (2) identifying relevant studies; (3) selecting the studies; (4) charting the data; and (5) collating, summarising, and reporting the results.

**Results:**

We will report our findings in accordance with the guidance provided in the Preferred Reporting Items for Systematic Reviews and Meta-Analyses Protocols (PRISMA-P) statement.

**Discussion:**

The review will generate the most up-to-date evidence and identify gaps in literature in addition to informing future research on how SHGs can help address SRH and HIV outcomes among FSWs in SSA. Additionally, the scoping review can potentially inform a subsequent systematic review.

## Introduction

Globally, female sex workers (FSWs) are 30 times more likely to acquire HIV than general population women ^1^. High numbers of sexual partners, inability to control prevention measures ^2^, stigma that fuels violence and, criminalisation of sex work, combine to reduce FSWs’ exposure to services and support, which in turn, increases their vulnerabilities to HIV and other sexually transmitted infections (STIs) ^3– 5^. A systematic review of community empowerment and involvement of FSWs in targeted sexual and reproductive health (SRH) interventions in Africa found that FSWs aged 15 to 49 years were at a higher risk of SRH morbidity, violence and discrimination compared to same age general population women ^6^.

In sub-Saharan Africa (SSA), the epicentre of the HIV epidemic ^7^ the burden of HIV and other STIs among FSWs is disproportionately high ^8^. For example, in Zimbabwe, HIV prevalence was estimated at 48% among FSWs in 2022 compared to 11% among the general population ^9^. It is noteworthy that sex work is an important feature of the transmission dynamics of HIV within early, advanced and regressing epidemics in SSA ^10^.

FSWs’ vulnerabilities complicate their ability to prevent or manage HIV; therefore, they need a more tailored approach to address the condition. Self-help groups (SHGs), where individuals with commonalities come together to support each other, are potentially impactful ^11^. The concept of the SHG as a catalyst for change was pioneered in India in the early 1980s ^6^. It emphasised high levels of group ownership, control and management concerning goals, processes and outcomes ^6^. According to Brody *et al.* ^12^, when individuals come together to take action towards overcoming obstacles and attaining social change, individual and/or collective empowerment can occur.

Past research has shown that SHGs, along with other community mobilisation and structural interventions, can empower FSWs to address their specific vulnerabilities ^13^. A study in India highlighted the impact of SHGs among this group when FSWs who attended SHGs demonstrated higher HIV knowledge, accessed services more, and were more likely to turn away clients who refused to use condoms, compared to those that did not attend SHGs ^14^. However, there is a lack of substantial evidence on how SHGs can address health-related outcomes of FSW in the SSA context. The proposed review seeks to explore whether and how SHGs improve FSWs’ SRH and HIV outcomes in sub-Saharan Africa.

SHGs may stimulate empowerment and increase sense of control. Among the theories that explain how SHGs influence empowerment is the integrated empowerment framework for FSWs, which combines various elements and strategies aimed at empowering individuals or communities ^15^. It integrates multiple aspects of empowerment to achieve meaningful and sustainable outcomes ^15^. The three main domains of this framework are power within, power with (others), and power over (resources) ^15– 17^. Briefly, power within implies the means by which FSWs develop their self-esteem, confidence and consciousness of the sources of their HIV vulnerability ^18– 20^, power with others entails their ability to effectively address power imbalances and achieve social transformation through collective identity, trust and mutual support ^21– 24^ and power over resources is their ability to exert power over the resources ^16, 17, 22, 24^ which they need through some form of control.

The integrated empowerment framework for FSWs can be applauded for its emphasis on promoting community mobilisation and economic empowerment by focusing on the three important domains of power that it proposes. However, there seems to be an overlap between the third domain (power over resources) and the other two domains (power within and power with others). One cannot talk about power over resources without reference to power within and power with others. A potential shortcoming of this framework is its disregard of FSWs’ disadvantaged position of power (various intersecting vulnerabilities in a world of unending inequalities) and not looking at other structural barriers and resource constraints that impact on FSWs as a unique group. My approach will borrow from some components of the framework ^15^.

## Methods

The main objective of the proposed scoping review is to explore a body of literature to identify what is known about SHGs in relation to addressing FSWs’ SRH and HIV outcomes in SSA. A preliminary search of MEDLINE, the Cochrane Database of Systematic Reviews and *JBI Evidence Synthesis* was conducted and no current or underway systematic or scoping reviews focusing on this topic were identified.

### Review question

*The scoping review will seek to answer the following questions:*

1. Do self-help groups improve sexual and reproductive health and HIV outcomes such as family planning and condom use, HIV/STI knowledge and prevention, HIV testing, treatment, and adherence, sexual self-efficacy, gender-based violence education, SRH knowledge/education, among female sex workers in sub-Saharan Africa?
  a. If so, what are the mechanisms through which SHGs improve these outcomes?
  b. What gaps exist in the evaluation of SHGs and SRH and HIV outcomes among FSWs in SSA?

### Eligibility criteria

The Population–Concept–Context (PCC) framework ^25^ will be used to develop the research objective(s) and question(s) to inform inclusion and exclusion criteria and consequently, the literature search strategy.

### Population

Female sex workers in SSA will be included. The proposed review will consider FSWs as women who receive money and/or goods or favours in exchange for sex. Transactional sex relationships will also be considered as sex work, even if participants do not self-identify as sex workers. The review will be restricted to studies conducted within SSA between 1 January 2000 and 30 September 2024. This period was chosen because it allows the search to include relevant articles within the SSA context considering SHGs are relatively new within this setting compared to others such as South Asia where they have a longer and institutionalised history ^26^. This period also considers that there was no similar scoping review during that time.

### Concept

The scoping review will explore the concepts of SHGs and sexual and reproductive health and HIV outcomes of FSWs.

### Context

Research studies conducted in sub-Saharan Africa.

### Types of sources

The scoping review will consider studies that meet the inclusion criteria. Quantitative data to be extracted will include characteristics of the study population, the proportion of the study population that consisted of FSWs, sample size, year of data collection, study location, sampling strategy and age range of the participants. The review will also consider qualitative studies that report on grounded theory, ethnography and qualitative descriptions.

### Study design

We will employ the framework developed by Arksey and O’Malley ^27^, which Levac *et al.* ^28^ and Colquhoun *et al.* ^29^ expanded on, and Peters ^30^ further outlined in the Joanna Briggs Institute Manual (2020 version). The stages of the review include: 1) identifying the research question, 2) identifying relevant studies, 3) selecting studies, 4) charting the data, and 5) collating, summarising, and reporting the results ^27^.

### Stage 1: Identifying the research question

We will use the Population–Concept–Context (PCC) framework ^25^ to identify the main concepts in the primary review question and to inform the search strategy. The primary review question will be, ‘Do self-help groups improve sexual and reproductive health and HIV outcomes among female sex workers in sub-Saharan Africa?’. A sub-question to this will be ‘If so, what are the mechanisms through which SHGs improve these outcomes?’ and another sub-question will be ‘What gaps exist in the evaluation of SHGs and SRH and HIV vulnerabilities among FSWs in SSA?’. These questions will enable us to map the range of relevant literature around these aspects and inform the direction of future research.

### Stage 2: Identifying relevant studies

***Search strategy and information sources***

Identification of studies relevant to this review will be achieved by searching electronic databases of the published literature which will include Medline, Global Health and CINAHL databases. The proposed search strategy is outlined in Table 1:

**Table 1.** Search Strategy.

Concept	Search terms
Empowerment groups	“Self help group*” OR “self-help group*” OR SHG* OR “collective*” OR “empowerment group*” OR “community group*” OR “group based activit*” OR “savings group*” OR “support group*” OR “mukando” OR “psycho social support group*” OR “peer support group*” OR “support system*” OR “safety net*” OR “morale booster*”
Sexual and reproductive health	“Sexual and reproductive health” OR “SRH” OR “reproductive health” OR “health outcome*” OR “sexual health” OR “health access” OR “access to health” OR “clinic uptake” OR “unintended pregnanc*” OR “STI*” OR “gender based violence*” OR “GBV” OR “human papilloma virus” OR “HPV” OR “safe sex” OR “family planning” OR “cervical cancer” OR “safe abortion”
HIV	“HIV” OR “hiv-1*” OR “hiv-2*” OR “hiv1” OR “hiv2” OR “HIV infect*” OR “human immunodeficiency virus” OR “human immuno-deficiency virus” OR “human immune-deficiency virus” OR “acquired immunodeficiency syndrome” OR “acquired immuno-deficiency syndrome” OR “acquired immune-deficiency syndrome” OR “HIV Infection*”
Female sex worker	“Female sex worker*” OR "FSW” OR “sex worker*” OR “prostitute*” OR “thigh vendor*” OR “transactional sex*” OR “bar maid*” OR “sex work*” OR “girls selling sex” OR “women selling sex” OR “female* selling sex” OR “young women selling sex” OR “transact* sex” OR “exchang* sex” OR “sell* sex” OR “sold sex” OR “trad* sex” OR “commercial sex” OR "escort" OR "hooker*" OR "streetwalker*" OR "whore" OR "hustler*" OR "woman of the street*" OR "bawd" OR "call girl*" OR "courtesan" OR "drab*" OR "tart*" OR "harlot*" OR "slut*"
Sub-Saharan Africa	“Africa, south of the Sahara” OR “sub-Saharan Africa” OR “Angola” OR “Benin” OR “Botswana” OR “Burkina Faso” OR “Burundi” OR “Cameroon” OR “Cape Verde” OR “Central African Republic” OR “CHAD” OR “Comoros” OR “Congo” OR “Congo Democratic Republic” OR “Djibouti” OR “Equatorial Guinea” OR “Eritrea” OR “Ethiopia” OR “Gabon” OR “Gambia” OR “Ghana” OR “Guinea” OR “Guinea-Bissau” OR “Cote d'Ivoire” OR “Ivory Coast” OR “Kenya” OR “Lesotho” OR “Liberia” OR “Madagascar” OR “Malawi” OR “Mali” OR “Mozambique” OR “Namibia” OR “Niger” OR “Nigeria” OR “Sao tome and Principe” OR “Rwanda” OR “Senegal” OR “Seychelles” OR “Sierra Leone” OR “Somalia” OR “South Africa” OR “South Sudan” OR “Sudan” OR “Swaziland” OR “Tanzania” OR “Togo” OR “Uganda” OR “Zambia” OR “Zimbabwe”

Grey literature will also be accessed; specifically, the Joint United Nations Programme on HIV/AIDS (UNAIDS) best practices and UNAIDS website, the World Health Organisation toolkit for FSWs and the reference lists from key articles, will be searched for relevant articles.

Search terms will be determined with input from the research team, research collaborators and knowledge users. The search strategy will be developed by the researcher with input from an experienced research librarian. Searches will be limited to literature published in English only and literature published before 2000 will be excluded so that the most recent literature is captured. We do not have resources to analyse studies written in other languages, and we recognise that this is a potential limitation of our review. Search results will be downloaded and imported into Endnote 20. After removing duplicates in Endnote 20, the articles will be exported to Covidence, a collaborative software.

### Stage 3: Study selection

Once the articles have been imported into Covidence, duplicates will be removed again. The review process will consist of two levels of screening: (1) a title and abstract review and (2) full-text review. The first author (GM) will do the first and second level of screening and a co-reviewer (ON) will do first level screening only. The criteria will be tested on a sample of abstracts prior to beginning the abstract review to ensure that they are robust enough to capture any relevant articles. Any articles that are deemed relevant by either or both reviewers will be included in the full-text review. In the second step, the first author will assess the full-text articles to determine if they meet the inclusion/exclusion criteria. The second reviewer will review 10% of the full text articles that would have been randomly selected for them. To determine inter-rater agreement, Covidence software will be used. Any discordant full-text articles will be reviewed a second time and further disagreements about study eligibility at the full-text review stage will be resolved through discussion with a more senior researcher (WM) until full consensus is obtained.

***Quality appraisal***

Quality appraisal will be conducted using the Critical Appraisal Skills Programme (CASP) tool ^31^, Cochrane Risk of Bias 2 (RoB 2) tool ^32^ and Risk Of Bias In Non-randomised Studies of Interventions (ROBINS-I) assessment tool ^33^, depending on a study’s design and methodology.

### Stage 4: Data charting

Data charting refers to ‘synthesising and interpreting data by sifting, charting, and sorting material according to key issues and themes’ (Arksey & O’Malley, 2005, p. 26). A data collection instrument will be developed by the first author to confirm study relevance and to extract study characteristics. Following Levac’s *et al.* ^28^ recommendations, we will develop a standard Excel data charting form that we will populate with key study characteristics. Study characteristics to be extracted will include, but not be limited to: publication year, publication type (e.g. original research), study design and country. This form will be reviewed and pretested by the reviewers before implementation to ensure that the form is capturing the information accurately. Data abstraction will be conducted in duplicate with two reviewers independently extracting data from all included studies. To ensure accurate data collection, each reviewer's independent abstracted data will be compared, and any discrepancies will be further discussed to ensure consistency between the reviewers. The data will be compiled in a single excel spreadsheet in Microsoft Excel software for validation and coding.

## Results

### Stage 5: Collating, summarising, and reporting the results

Following Levac’s *et al.* ^28^ suggestions, we will synthesise our data in three phases. First, we will analyse and summarise all charted data about study characteristics (see examples in the previous section) using easy-to-understand descriptive statistics. We will use thematic analysis to analyse and summarise all charted data relating to key issues and themes vis-à-vis the objectives of our scoping review. In the second phase, we will report our results. We will present quantitative results using tables and/or graphs and qualitative findings using key themes. We will conduct our reporting iteratively and continuously adjust our reporting scheme. Finally, we will discuss the meanings of our findings, including implications for policies and practice and further research, in terms of the objectives of our scoping review ^28, 29^.

## Discussion

Self-help groups (SHGs) have facilitated change in terms of social, psychological, economic and political outcomes ^12^. They facilitate empowerment which, in turn, makes it possible for marginalised groups to access what they normally cannot easily access in the absence of that empowerment. Brody *et al.* ^12^, asserts that women’s empowerment is considered an essential component of international development and poverty reduction. However, it is important to acknowledge that SHGs are not without challenges, as some interventions experience negative outcomes ^12^ such as barriers to participation including economic and social standing ^34, 35^, stigma as a result of participation ^12^, disappointment as a result of not getting expected outcomes and also mistrust and corruption in the groups ^34^, just to mention a few.

Research conducted in India and SSA suggests that SHGs can facilitate women’s empowerment ^12, 36^. Nevertheless, there is a lack of understanding regarding the potential of SHGs to enhance HIV and SRH outcomes among FSWs in SSA. Given that FSWs constitute a unique and vulnerable group, there is need to explore whether SHG membership is linked to improved HIV and SRH outcomes among this population. Community mobilisation has been found to be effective as an approach that helps FSWs and, SHGs are one approach of building collective efficacy ^37^. The proposed review aims to not only establish this link, but also to identify the mechanisms through which SHGs can enhance HIV and SRH outcomes among FSWs, while also addressing the existing gaps in the evaluation of this relationship.

The findings of this review will complement other existing strategies for addressing HIV and SRH challenges among FSWs in SSA. The review will primarily describe what is known rather than generate new knowledge. The focus is mainly on mapping the breadth of studies rather than the depth of information, describing what is known rather than necessarily contributing new knowledge ^38^. The strength of this review lies in involvement of highly qualified reviewers who will engage in constant collaboration and triangulation of findings during the data extraction phase.

We will share the results of this scoping review with diverse stakeholders through various channels including peer-reviewed publications, conferences, stakeholder meetings with organisations working with FSWs and other partners implementing programmes that seek to address SRH and HIV challenges of FSWs in SSA, with the aim of informing and potentially influencing future interventions and evaluations in this area.

## Conclusion

Here, we present a protocol to scope existing research to understand SHGs and how they can improve FSWs’ HIV and SRH outcomes in SSA. The scoping review will contribute to the existing literature by addressing a dearth of knowledge on the specific link between SHG membership and HIV and SRH outcomes among FSWs in SSA. By exploring this relationship and identifying the mechanisms through which SHGs can enhance HIV and SRH outcomes, the review will provide valuable insights for the development of tailored interventions for this vulnerable population.

## Ethics and consent

Ethical approval and consent were not required.

## Data Availability

No data associated with this article. ***Reporting guidelines*** Open Science Framework: PRISMA-P checklist for “Do self-help groups improve sexual and reproductive health and HIV outcomes among female sex workers in sub-Saharan Africa? A scoping review protocol”. https://doi.org/10.17605/OSF.IO/JATC2 ^39^ Data are available under the terms of the Creative Commons Zero "No rights reserved" data waiver (CC0 1.0 Public domain dedication).
