# Supplementary material for: Exploring the contribution of self-help groups to sexual and reproductive health and HIV outcomes for female sex workers in sub-Saharan Africa: A scoping review protocol
Source: Wellcome Open Res. 2025 Jun 16;9:535. Originally published 2024 Sep 19. [Version 2] doi: 10.12688/wellcomeopenres.23002.2 (PMC12648005; doi:10.12688/wellcomeopenres.23002.2)
Supplement: Peer review history

# Reviewer report: Reviewer response for version 2

**DOI:** 10.21956/wellcomeopenres.26875.r138838

**Published:** 2025-11-25

**Authors:** Penelope Phillips-Howard

**Affiliations:** 1 Liverpool School of Tropical Medicine, Liverpool,, UK

This is a protocol paper, describing the topic of interest, the contribution of self-help groups to sexual and reproductive heath and HIV outcomes for female sex workers in sub-Saharan Africa. It provides a good rationale for needing a scoping review, and describes the underlying framework. It summarizes clearly the step-wise approach that will be used in the methods. Sufficient detail is given to allow this work to be replicated. It is well written, and with the additional changes made in amendment 1, it adequately describes the search methodology, and definitions.  It is a balanced discussion which is always tricky given it is a protocol paper. I have no concerns about indexing.

Is the study design appropriate for the research question?

Yes

Is the rationale for, and objectives of, the study clearly described?

Yes

Are sufficient details of the methods provided to allow replication by others?

Yes

Are the datasets clearly presented in a useable and accessible format?

Not applicable

Reviewer Expertise:

sexual and reproductive health of vulnerable populations in sub Saharan Africa, including female sex workers

I confirm that I have read this submission and believe that I have an appropriate level of expertise to confirm that it is of an acceptable scientific standard.

# Reviewer report: Reviewer response for version 1

**DOI:** 10.21956/wellcomeopenres.25331.r121593

**Published:** 2025-04-11

**Authors:** Dilini Mataraarachchi

**Affiliations:** 1 Keele University, Keele, UK

This is an interesting research area, and the authors intend to do a scoping review to identify how self-help groups can reduce the STI risk of female sex workers in Africa.

 The authors have discussed the importance of self-help groups when sending health messages to the FSW using relevant literature. However, why this is conducted as a scoping review rather than a systematic review needs to be justified.

 In the methodology section, authors have to state how they developed the search strategy and who was involved. Also please include a section for results analysis and synthesis. Furthermore, I suggest adhering to the COCHRANE quality appraisal tools when assessing the quality of studies.

 Furthermore, please mention whether you have registered this protocol in PROSPERO and if yes mention the protocol registration number.

 When all these comments are addressed the article is fit for publishing in this journal.

Is the study design appropriate for the research question?

Yes

Is the rationale for, and objectives of, the study clearly described?

Yes

Are sufficient details of the methods provided to allow replication by others?

Partly

Are the datasets clearly presented in a useable and accessible format?

Yes

Reviewer Expertise:

adolescent health, sexual and reproductive health, gender studies

I confirm that I have read this submission and believe that I have an appropriate level of expertise to confirm that it is of an acceptable scientific standard, however I have significant reservations, as outlined above.

# Response

**Published:** 2025-06-05

**Authors:** Gracious Madimutsa

**Affiliations:** Centre for Sexual Health and HIV/AIDS Research Zimbabwe, Harare, Harare Province, Zimbabwe

This is an interesting research area, and the authors intend to do a scoping review to identify how self-help groups can reduce the STI risk of female sex workers in Africa.

**Response:** Thank you for these positive comments.  

 The authors have discussed the importance of self-help groups when sending health messages to the FSW using relevant literature. However, why this is conducted as a scoping review rather than a systematic review needs to be justified.

**Response:** Because our study objective is broad, we felt that a scoping review is more appropriate than a systematic review. The scoping review may inform a subsequent systematic review. We now state this.  

 In the methodology section, authors have to state how they developed the search strategy and who was involved.

**Response:** We now state this.  

 Also please include a section for results analysis and synthesis. Response: Analysis and synthesis of results will be done in stage 5: Collating, summarising, and reporting the results.   Furthermore, I suggest adhering to the COCHRANE quality appraisal tools when assessing the quality of studies.

 Response: We no longer plan to conduct quality appraisal as this is not a key concern of a scoping review methodology (Arksey & O’Malley, 2005). We recognise that this is a potential limitation of our review. We may, however, suggest recommendations on how future studies can address quality issues based on the findings of our scoping review.   We have now deleted the section on Quality Appraisal.  

 Furthermore, please mention whether you have registered this protocol in PROSPERO and if yes mention the protocol registration number.

 Response: We have registered the protocol on Open Science Framework. Available: https://doi.org/10.17605/OSF.IO/JATC2

# Reviewer report: Reviewer response for version 1

**DOI:** 10.21956/wellcomeopenres.25331.r100263

**Published:** 2024-10-27

**Authors:** Chido Dziva Chikwari

**Affiliations:** 1 London School of Hygiene & Tropical Medicine, London, UK

Review Comments

 This manuscript is a scoping review protocol for self-help groups to improve HIV and SRH outcomes among female sex workers in SSA. The authors propose a review for literature from 2000-2024 and aim to use the Population–Concept–Context (PCC) framework.

 Overall, the manuscript is comprehensive and well written. There are no major comments or revisions.

 The findings will be of importance for consideration FSW programming.

 General comments

- It is not clear why a scoping rather than systematic review has been chosen
- There are some instances where first person singular pronouns are used and others where first-person plural pronouns are used; it will be important to read through the manuscript and ensure consistency
- Where acronyms have already been defined e.g. SRH and SSA they are sometimes written in full thereafter, this may not be necessary

Is the study design appropriate for the research question?

Yes

Is the rationale for, and objectives of, the study clearly described?

Yes

Are sufficient details of the methods provided to allow replication by others?

Yes

Are the datasets clearly presented in a useable and accessible format?

Not applicable

Reviewer Expertise:

SRH, HIV, STIs, Adolescent Health, Epidemiology.

I confirm that I have read this submission and believe that I have an appropriate level of expertise to confirm that it is of an acceptable scientific standard.

# Response

**Published:** 2025-06-05

**Authors:** Gracious Madimutsa

**Affiliations:** Centre for Sexual Health and HIV/AIDS Research Zimbabwe, Harare, Harare Province, Zimbabwe

Reviewer 1 This manuscript is a scoping review protocol for self-help groups to improve HIV and SRH outcomes among female sex workers in SSA. The authors propose a review for literature from 2000-2024 and aim to use the Population–Concept–Context (PCC) framework.   Overall, the manuscript is comprehensive and well written. There are no major comments or revisions. The findings will be of importance for consideration FSW programming. Response: Thank you for these positive comments.   General comments

- It is not clear why a scoping rather than systematic review has been chosen

Response: Because our study objective is broad, we felt that a scoping review is more appropriate than a systematic review. The scoping review may inform a subsequent systematic review. We now state this.

- There are some instances where first person singular pronouns are used and others where first-person plural pronouns are used; it will be important to read through the manuscript and ensure consistency

Response: Thank you for this observation. We have ensured consistency throughout.

- Where acronyms have already been defined e.g. SRH and SSA they are sometimes written in full thereafter, this may not be necessary

Response: Thank you. We have made the necessary changes.
